# Testing of VKORC1 and CYP2C9 alleles to guide warfarin dosing: Test Category: Pharmacogenomic (Treatment)

**DOI:** 10.1371/currents.RRN1155

**Published:** 2010-09-14

**Authors:** Daurice Grossniklaus

**Affiliations:** National Office of Public Health Genomics, CDC

## Abstract

Warfarin is an oral anticoagulant that is widely prescribed to prevent thromboembolic events in persons at increased risk. The optimal dose is difficult to establish because it can vary 10-fold among individuals due to clinical and demographic factors. Testing for variants of the vitamin K epoxide reductase complex 1 (VKORC1) and cytochrome P450 2C9 (CYP2C9) genes has been proposed for use in guiding the initial dose of warfarin, thus achieving optimal dosing more quickly and with lower risk of bleeding.

## **Clinical Scenario**

Pharmacogenetic testing to guide warfarin dose selection for individuals at risk of a thromboembolic event with the goal of shortening the time required to achieve a stable, effective dose and minimizing the risk of adverse effects.[1]

## **Test Description**

Analysis of multiple single nucleotide polymorphisms (SNPs) in *VKORC1* and *CYP2C9*.

## **Public Health Importance**

Over 31 million warfarin prescriptions were dispensed in 2004. Bleeding from warfarin use is a common adverse event and can cause substantial morbidity and mortality.[2]

## **Published Reviews, Recommendations and Guidelines**

**Systematic evidence reviews**

    Rapid ACCE review [3] from 2008 includes a discussion of ELSI issues and identifies knowledge gaps.

Recommendations by independent group*

    None identified

**Guidelines by professional group**

    2008 American College of Medical Genetics (ACMG) policy statement: "There is insufficient evidence, at

    this time, to recommend for or against routine *CYP2C9* and *VKORC1* testing in warfarin-naive patients."[4]

2008 American College of Chest Physicians guideline statement: "We suggest against pharmacogeneitc-based dosing until randomized data indicate that it is beneficial."[5]

Some additional guidelines and recommendations can be found at Centers for Medicare & Medicaid Services Decision Memo for Pharmacogenomic Testing for Warfarin Response (CAG-00400N)(dated 8/3/2009): 

http://www.cms.gov/mcd/viewdecisionmemo.asp?from2=viewdecisionmemo.asp&id=224

* independent groups include the US Preventive Services Task Force (USPSTF) and Evaluation of Genomic *Applications in Practice and Prevention (EGAPP) Working Group.*

## **Evidence Overview**

***Analytic Validity*:**

Test accuracy and reliability in identifying alleles at multiple SNPs (analytic sensitivity and specificity).

- Based on findings from a ACCE evidence review and ACMG policy statement:

- Limited data on analytic validity are available from laboratories performing these tests.[4]
- High analytic sensitivity and specificity is expected for testing of common *CYP2C9* alleles.[3] [4]

- Few data are available for evaluation on analytic sensitivity and specificity of testing for *VKORC1* alleles.[3]

- College of American Pathology (CAP)/American College of Medical Genetics proficiency testing program for warfarin pharmacogenetic testing may improve access to analytic validity data.[3] [4] http://www.cap.org/apps/docs/proficiency_testing/surveys_catalog/2010_surveys_catalog.pdf#page=214

***Clinical Validity*:**Test accuracy and reliability in predicting appropriate warfarin dose (predictive value).

- Published studies have examined the relationship of *CYP2C9* alleles to warfarin dose.[6]

HuGE Navigator: query "warfarin and *CYP2C9* *"*

- Published studies have examined the relationship of *VKORC1* alleles to warfarin dose.[6]   

HuGE Navigator: query "warfarin and *VKORC1*"

- The ACCE review and ACMG guideline reported that there is evidence that CYP2C9 and VKORC1 variants are correlated with the stable warfarin dose. There is limited evidence for an association between CYP2C9 and severe bleeding events, and an absence of evidence for bleeding events associated with VKORC1.[3] [4]  

Recent additions to the literature:

- A study designed to compare a clinical algorithm versus a pharmacogenetic algorithm using INR values (day 4 or 5 of treatment), clinical factors and genotype to predict warfarin dose. In the derivation set (N=969), the clinical algorithm had an coefficient of determination R(2) of 48% and the pharmacogenetic algorithm had an R(2) of 63% in predicting warfarin dose. In independent validation sets, the clinical algorithm had an R(2) of 26-43% and the pharmacogenetic algorithm had an R(2) of 42-58% in predicting warfarin dose.[7]
- A retrospective cohort study designed to examine the accuracy of pharmacogenetic warfarin dosing algorithms in predicting warfarin dose. Data from 71 adult patients at an outpatient anticoagulation clinic on a stable, therapeutic warfarin dose were included in the analysis. Six pharmacogenetic warfarin dosing algorithms and a 5 mg fixed dose approach were evaluated. The algorithms published by Gage et al. 2008 and the IWPC 2009 were the most accurate in predicting warfarin dose in the study population.[8]
- A study designed to compare the International Warfarin Pharmacogenetics Consoritum (IWPC) algorithm versus a clinical algorithm in cohort of Japanese patients (n=200). The purpose was to determine the percentage of Japanese patients for whom the predicted dose deviated by less than 7mg/week from the actual dose. The IWPC algorithm identified a larger percentage of patients to achieve the target INR than did the clinical algorithm.[9]
- A study by the International Warfarin Pharmacogenetics Consortium reported a comprehensive assessment of the influence of six *VKORC1* SNPs and haplotypes on warfarin dose prediction in a cohort of Asians (n=1103), blacks (n=670), and whites (n=3113).[10]

- VKORC1-1639G>A and 1173C>T individually explained the greatest variance in warfarin dose across the three racial groups. Including additional *VKORC1* SNPS and haplotypes did not further improve warfarin dose prediction.
- *VKORC1 *explained greater variability in warfarin dose among whites than in Asians or blacks, a finding explained largely by race-specific differences in the frequency of the -1639 A and 1173T alleles.  

- A study designed to examine the effect of *CYP2C9* and *VKORC1* genotypes on rate of International Normalized Ratio (INR) increase, anticoagulation maintenance, risk of over anticoagulation, and change in dose over 30 days.[11]
  - The *VKORC1* variant genotype (with/without the *CYP2C9* variant genotype) was associated with higher risk of over anti-coagulation in European Americans but not African Americans. 
  - The risk of minor hemorrhage was not influenced by either *CYP2C9* or *VKORC1* genotype.
  - Over the first 30 days of therapy, *CYP2C9* and *VKORC1* genotypes explained 6.3% of the variance in dose change.

- The International Warfarin Pharmacogenetics Consortium reported that an algorithm based on genetic and clinical data performed better than a purely clinical algorithm or a fixed-dose approach in estimating the appropriate initial dose of warfarin.[1]
- Results of a randomized clinical trial suggested that an algorithm including *CYP2C9* and *VKORC1* along with clinical factors improved accuracy and efficiency of warfarin dose when compared with an empirical protocol; however, there was no difference in the primary endpoint, the proportion of out-of-range clotting times.[12]
 
- ***Clinical Utility*: **
- Net benefit of test in improving health outcomes.

- The ACCE review reported controlled trials that evaluate the net effect of testing for *CYP2C9 *and *VKORC1* alleles on health outcomes in persons treated with warfarin have not been conducted.[3]
- Recent additions to the literature:

- A national, prospective comparative effectiveness study was designed to examine the 6-month incidence of hospitalization (event-free time) in patients receiving warfarin genotyping (n=896) compared to a matched historical control group (n=2,688). To evaluate possible temporal changes in general clinical practice, the researchers compared the hospitalization rates for two external control groups (an external concurrent control group and an external historical control group).[13]

- Adjusted hospitalization rates based on the intention-to-treat analysis showed that the genotyped group had a 31% lower rate of all-cause hospitalizations and a 28% lower rate of hospitalizations for bleeding or thromboembolism compared to the matched historical control group during the 6 month follow up period. There were no significant differences in the adjusted hospitalization rates between the two external control groups.[13]
- A per-protocol analysis was conducted in which only events occurring after genotyping were counted for patients in the genotyped group. Adjusted hospitalization rates based on the per-protocol analysis showed that the genotyped group had a 33% lower rate of all-cause hospitalizations and a 43% lower rate of hospitalizations for bleeding or thromboembolism compared to the matched historical control group during the 6 month follow up period. There were no significant differences in the adjusted hospitalization rates between the two external control groups.[13]

-  A study designed to compare gene based warfarin dosing versus standard of care practices in an orthopedic surgery population. Adults (n=229) undergoing elective total hip and knee arthroplasty  and receiving warfarin under the direction of a dedicated anticoagulation services team were enrolled. The primary endpoint was the reduction in the incidence of adverse events; additional endpoints included time to first therapeutic International Normalized Ratio (INR), time to first supratherapeutic INR; and the percent of INR determinations that fell below, within or above the therapeutic range. Endpoints did not achieve statistical significance.[14]

- Four published dosing algorithms and a prediction model incorporating the CYP2C9*2, CYP2C9*3, and *VKORC1-1639* polymorphisms were evaluated in two independent datasets (total n=1095).[15]

- The four pharmacogenetic-based dosing algorithms performed similarly in the small, white-only dataset and the large, ethnically diverse dataset.
- The International Warfarin Pharmacogenetics Consortium algorithm performed best overall for the two datasets combined when comparing the percent of patients whose predicted dose was within 20% of the actual dose.

- A study of patients with nonvalvular atrial fibrillation examining the cost-effectiveness of genotype guided dosing compared to standard of care for initiating warfarin treatment.  Study design was Markov state transition decision model, and data sources included MEDLINE searches and bibliographies from relevant articles. The outcome measures were quality-adjusted life years (QALYs) and costs in US dollars. The authors concluded that based on current data and cost of testing, there is a 10% chance that genotype guided dosing is likely to be cost effective for nonvalvular artial fibrillation patients.[16]

- The Center for Medicare and Medicaid Services (CMS) does not generally reimburse costs, but it does provide coverage for individuals enrolled in appropriate clinical trials designed to examine clinical utility of genetic testing for Warfarin dosing. See CMS decision memo.  

  

## **Links**

**U.S, Food and Drug Administration:** Table of Valid Genomic Biomarkers in the Context of Approved Drug Labels; Search FDA 510(k) database

**ClinicalTrials.gov:** Warfarin and CYP2C9, Warfarin and VKCORC1   

**PharmGKB** **:** warfarin, International Warfarin Pharmacogenetics Consortium

Last updated: April 12, 2010 
